# Relationships Between Vitamin D Status and PTH over 5 Years After Roux-en-Y Gastric Bypass: a Longitudinal Cohort Study

**DOI:** 10.1007/s11695-020-04582-5

**Published:** 2020-04-18

**Authors:** Stephen Hewitt, Jon Kristinsson, Erlend Tuseth Aasheim, Ingvild Kristine Blom-Høgestøl, Eirik Aaseth, Jørgen Jahnsen, Erik Fink Eriksen, Tom Mala

**Affiliations:** 1 Department of Endocrinology, Morbid Obesity and Preventive Medicine, Oslo University Hospital HF, Aker, P.O. Box 4950 Nydalen, 0424 Oslo, Norway; 2 Institute of Clinical Medicine, University of Oslo, 0450 Oslo, Norway; 3 Department of Gastrointestinal Surgery, Oslo University Hospital, P.O. Box 4950, Nydalen, 0407 Oslo, Norway; 4 Department of Global Health and Documentation, Norwegian Directorate of Health, P.O. Box 220, Skøyen, 0213 Oslo, Norway; 5 Department of Medicine, Innlandet Hospital Trust, Elverum, Norway; 6 Department of Gastroenterology, Akershus University Hospital, 1474 Lørenskog, Norway

**Keywords:** Bariatric surgery, Morbid obesity, Vitamin D, Calcium, Parathyroid hormone, Bone turnover

## Abstract

**Purpose:**

Secondary hyperparathyroidism (SHPT) after obesity surgery may affect bone health. Optimal vitamin D levels have not been established to prevent SHPT postoperatively. We investigated whether SHPT differed across threshold levels of serum 25-hydroxyvitamin D (S-25(OH)D) from 6 months up to 5 years after Roux-en-Y gastric bypass (RYGB).

**Materials and Methods:**

We included 554 patients at follow-up 5 years postoperatively. Blood samples were analysed for S-25(OH)D, ionized calcium (iCa) and parathyroid hormone (PTH) during follow-up.

**Results:**

PTH and prevalence of SHPT increased from 6 months to 5 years postoperatively, while S-25(OH)D and iCa decreased (all *P* < 0.001). PTH and SHPT development are related with S-25(OH)D, and PTH differed between all subgroups of S-25(OH)D. SHPT occurred less frequently across all subgroups of S-25(OH)D ≥ 50 nmol/l during follow-up: odds ratio (OR) 0.44 (95% CI 0.36–0.54) in patients with S-25(OH)D ≥ 50 nmol/l, OR 0.38 (0.30–0.49) with S-25(OH)D ≥ 75 nmol/l and OR 0.19 (0.12–0.31) with S-25(OH) D ≥ 100 nmol/l, all compared with S-25(OH)D < 50 nmol/l. At 5 years, 208/554 patients (38%) had SHPT; SHPT was found in 94/188 patients (50%) with S-25(OH)D < 50 nmol/l, in 69/222 (31%) with S-25(OH)D 50–74 nmol/l, in 40/117 (34%) with S-25(OH)D 75–99 nmol/l and in 5/27 (19%) with S-25(OH)D ≥ 100 nmol/l. An interaction existed between S-25(OH)D and iCa. Bone alkaline phosphatase remained increased with SHPT.

**Conclusions:**

A significant relationship existed between S-25(OH)D and development of PTH and SHPT. The prevalence of SHPT was lower with threshold levels 25(OH)D ≥ 50 nmol/l and ≥ 75 nmol/l over the 5 years, and lowest with S-25(OH)D ≥ 100 nmol/l.

**Electronic supplementary material:**

The online version of this article (10.1007/s11695-020-04582-5) contains supplementary material, which is available to authorized users.

## Introduction

Obesity surgery provides effective weight loss in morbid obesity [1, 2]. Changes in gastrointestinal anatomy and physiology may also influence intestinal uptake and nutritional status, including for calcium and vitamin D [1–6]. Calcium and vitamin D are central in parathyroid hormone (PTH) regulation [7, 8]. Frequently, PTH is elevated after obesity surgery, and long-term studies indicate that PTH increases over time [4, 9–15].

Relationships of calcium and vitamin D with PTH should be explored further, as PTH may impact bone turnover and bone mineral density (BMD) postoperatively [14–24]. More focus on these are needed during follow-up. Supplementation of calcium and vitamin D is recommended after obesity surgery, but secondary hyperparathyroidism (SHPT) remains prevalent [22, 25]. Few studies have shown differences in prevalence of SHPT with 25-hydroxyvitamin D (S-25(OH)D) threshold levels higher than ≥ 50 nmol/l after obesity surgery [9, 20]. It could be that optimal vitamin D should be higher after obesity surgery than in other nonsurgical populations [7, 8, 24, 26].

We aimed to study the development of PTH and SHPT over 5 years after Roux-en-Y gastric bypass (RYGB), and relationships between these and different threshold levels of S-25(OH)D ≥ 50 nmol/l. We assessed whether the prevalence of SHPT would be lower among patients with higher S-25(OH)D up to 5 years postoperatively.

## Materials and Methods

### Patients and Study Design

This longitudinal observational cohort study was analysed prospectively, and the report was written to comply with the STROBE checklist [27].

Morbid obesity was defined as BMI ≥ 40 kg/m^2^, or BMI ≥ 35 kg/m^2^ with obesity-related comorbidities [28]. Obesity surgery was offered at Oslo University Hospital, Aker, after failed weight loss by other means. Laparoscopic RYGB was the preferred procedure in the period, with construction of a gastric pouch 25–30 ml, a 150-cm antegastric, antecolic alimentary limb and a 50-cm biliopancreatic limb [29, 30].

We aimed for minimum 500 patients in a population with high follow-up. Candidates were RYGB patients operated 2004–2009. They were evaluated preoperatively and postoperatively with weight, height and blood samples. Follow-up visits were after 6 weeks, 6 months, 1 year, 2 years, 3–4 years and 5 years. At 5 years, all were contacted by letter and eventually by telephone. Body weight was measured electronically (platform weight, Seca 635 0–300 kg, class III), height with wall-fixed steel measure and blood samples were drawn after overnight fast.

Patients with signed consent and valid PTH and S-25(OH)D at 5 years were candidates, while patients with primary hyperparathyroidism and elevated creatinine were excluded.

### Laboratory Analyses

Serum intact PTH (1–84), S-25(OH)D, ionized calcium (iCa) and bone specific alkaline phosphatase (B-ALP) were determined at the Hormone Laboratory, Oslo University Hospital, Aker. PTH was analysed by a chemiluminoimmunometric assay (Immulite 2000/2500, Siemens Health Care Diagnostics) (reference range 1.5–7.0 pmol/l, coefficient of variation (CV) 7%). S-25(OH)D was determined by radioimmunoassay (Dia-Sorin) (reference range 37–131 nmol/l, CV 14%), from September 2012 with liquid chromatography-mass spectrometry (LC-MS/MS) (identical reference range, CV 9%); measurements were comparable. Different methods were used for iCa: first, (a) CIBA Corning, instrument 634 Ca^2+^/pH Analyser (Bayer); from December 2005 to December 2007, (b) Rapidlab 348 pH/Blood Gas Analyser (Instru-Med) (both reference range 1.15–1.35 mmol/l, CV 1% and 2%, respectively); from January 2008 to January 2012, (c) Rapidlab 348 pH/Blood Gas Analyser (Instru-Med) (reference range 1.18–1.35 mmol/l); from February 2012, (d) Cobas b221 (Roche Diagnostics) (reference range 1.15–1.33 mmol/l, CV 2%). Reference for iCa was (c) with reference range 1.18–1.35 mmol/l. Values from (a) and (b) were transformed to (c) adding 0.015 mmol/l (differences between reference ranges). Similarly were values from (d) transformed to (c) adding 0.025 mmol/l. B-ALP was determined enzymatically after immune-extraction (Metra Biosystems) (reference range: women 12–31 U/l, men 15–41 U/l (CV 12%). Standard analyses included S-25(OH)D, iCa and PTH during follow-up, which were analysed sporadically preoperatively. Phosphate, magnesium, creatinine and total alkaline phosphatase (ALP) in plasma were analysed at the Central Laboratory, Oslo University Hospital, Aker, with a Modular (Roche) analyzer: Phosphate and magnesium were determined photometrically, creatinine enzymatically and ALP by an enzymatic calorimetric measurement.

### Definitions and Subgroups

SHPT was defined as PTH > 7.0 pmol/l with no elevation of iCa (i.e. ≤ 1.35 mmol/l). S-25(OH)D was grouped into 4 categories, < 50, 50–74, 75–99 and ≥ 100 nmol/l. S-25(OH)D < 50 nmol/l is commonly acknowledged as vitamin D deficiency (or insufficiency) [7, 8]. Calcium levels were grouped into 3 by normal reference range (iCa ≤ 1.23, 1.24–1.29, ≥ 1.30 mmol/l); all iCa values ≤ 1.23 mmol/l are termed in the “lower range”. Values below normal reference range (< 1.18 mmol/l) are termed “Low iCa”.

### Supplementation

Recommended daily supplements included one multivitamin (cholecalciferol 200 IU) and two combination tablets, each containing calcium carbonate 500 mg and cholecalciferol 400 IU. Compliance was defined by use of calcium ≥ 500 mg and vitamin D ≥ 600 IU minimum 5 days a week, noncompliance as less or no use. Supplements were adjusted to keep blood values within normal reference range, from 2012 to maintain S-25(OH)D ≥ 50 mmol/l, or S-25(OH)D ≥ 75 nmol/l in cases with SHPT [7, 8]. Our supplementation regimen also included oral iron (100 mg daily) and intramuscular vitamin B12 injections (1 mg per 3 months).

### Statistical Analyses

Statistical analyses were performed with IBM SPSS for Windows, version 25. Continuous and categorical variables were tested with *t* test and chi-square test as appropriate. Regression analyses were performed with linear mixed model, diagonal covariance matrices for PTH and B-ALP using individual repeated measurements, time-dependent covariates and random intercept. Variables were included in multivariate analyses of PTH with stepwise backward elimination of nonsignificant variables. Gender, age and BMI were included as covariates. We tested multiplicative interactions for S-25(OH)D, iCa and time on PTH, and PTH and time on B-ALP. B-ALP was adjusted for gender. Generalized estimating equations (GEE), unstructured covariance matrices were used for SHPT. Missing data were not imputed. We analysed two periods: from baseline to 6 months postoperatively and from 6 months to 5 years. The second period was the main focus, as S-25(OH)D and PTH were routinely assessed. Continuous variables are presented with means and standard deviations (± SD), categorical variables in percentages, and odds ratios (OR) and relative ratios (RR) with 95% confidence intervals (95% CI).

## Results

Of 823 operated, 584 (71%) attended 5-year follow-up. Included were 554 patients (67%), after exclusion of 4 with no signed consent, 10 with a suspicious primary hyperparathyroidism, 3 with elevated creatinine, and 13 with missing data of S-25(OH)D and PTH at 5 years. Follow-up period was 5.3 ± 0.4 years. Three patients had moved from our region and could not be contacted, and 7 had died during the 5 years. Table 1 summarizes preoperative characteristics.

**Table 1:** Preoperative characteristics (*N* = 554)

	*N*	Mean	SD
Women	384		
Age (y)	554	41.7	9.1
Weight (kg)	554	136	22
Height (cm)	554	172	10
BMI (kg/m^2^)	554	46.2	5.3
Blood samples			
PTH (pmol/l)	160	6.4	2.8
S-25(OH)D (nmol/l)	158	53	22
iCa (mmol/l)	245	1.25	0.04
Phosphate (mmol/l)	204	1.02	0.19
Magnesium (mmol/l)	247	0.81	0.07
Creatinine (μmol/l)	549	68	13
B-ALP (U/l)	44	26.3	6.6
SHPT	56	(35%)	

*BMI*, body mass index; *PTH*, parathyroid hormone; *S-25(OH)D*, serum 25-hydroxyvitamin D; *iCa*, ionized calcium; *B-ALP*, bone alkaline phosphatase; *SHPT*, secondary hyperparathyroidism

### The First 6 Months

BMI decreased 27% in the first 6 months from 46.2 ± 5.3 to 33.8 ± 5.1 kg/m^2^, PTH decreased from 6.4 ± 2.8 to 5.2 ± 2.5 pmol/l and prevalence of SHPT from 35 to 18% (all *P* < 0.001). S-25(OH)D and iCa were independently related with PTH development (*P* < 0.001).

PTH decreased in all subgroups of S-25(OH)D. Patients with S-25(OH)D < 50 nmol/l had highest PTH preoperatively and 6 months postoperatively, and they had the largest decrease in PTH (*P* < 0.001). PTH related positively with B-ALP (*P* < 0.001).

### Six Months to 5 Years Postoperatively

**Fig. 1:**
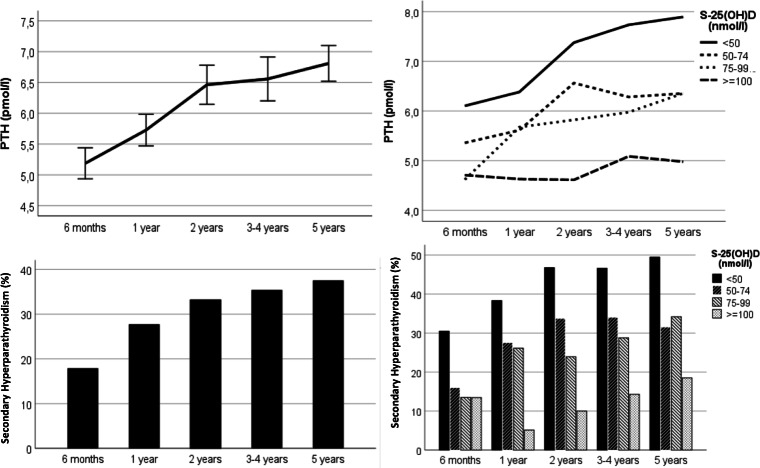
Observed development of parathyroid hormone (PTH) and secondary hyperparathyroidism (%) from 6 months to 5 years after Roux-en-Y gastric bypass (*N* = 554) to the left, and for subgroups of 25-hydroxyvitamin D (S-25(OH)D) to the right

Figure 1 illustrates development of PTH and SHPT from 6 months to 5 years postoperatively. PTH increased to 6.8 ± 3.5 pmol/l, and the prevalence of SHPT increased to 38%, while S-25(OH)D decreased to 59 ± 24 nmol/l, and iCa to 1.22 ± 0.04 mmol/l (all *P* < 0.001). BMI increased to 34.5 ± 6.2 kg/m^2^ (*P* = 0.005).

### Vitamin D, PTH and SHPT

S-25(OH)D was inversely related with development of PTH and occurrence of SHPT from 6 months to 5 years (P < 0.001), and PTH differed between all subgroups of S-25(OH)D (P < 0.001) (Fig. 1). Mean PTH response by change in vitamin D levels was.

$$ \Delta  \mathrm{PTH}\left(\mathrm{pmol}/\mathrm{I}\right)=-0.031\ast \Delta  25\left(\mathrm{OH}\right)\mathrm{D}\left(\mathrm{nmol}/\mathrm{I}\right) $$

expressing that each 1 nmol/l increase in S-25(OH)D related with a PTH decrease of 0.031 pmol/l during follow-up, and vice versa. From 6 months, PTH and prevalence of SHPT increased most with S-25(OH)D < 50 nmol/l (P < 0.001), but not significantly with S-25(OH)D ≥ 100 nmol/l.

**Fig. 2:**
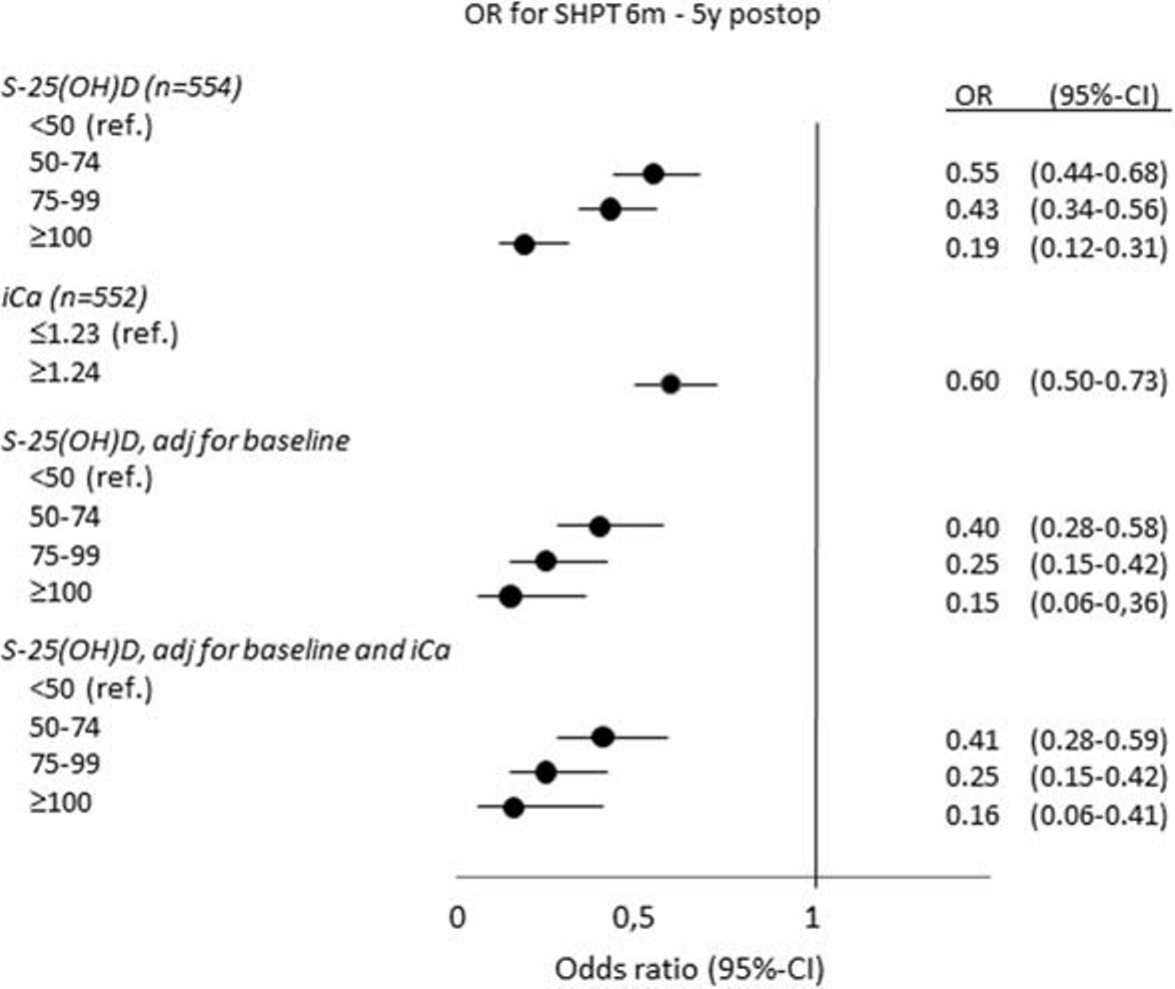
Odds ratio (OR) for secondary hyperparathyroidism (SHPT) from 6 months to 5 years postoperatively in 554 Roux-en-Y gastric bypass patients by categories of serum 25-hydroxyvitamin D (S-25(OH)D, nmol/l) and ionized calcium (iCa, mmol/l) compared with reference (ref.). S-25(OH)D was adjusted for time (y): OR 1.11 (1.02–1.22), with additional adjustments for baseline S-25(OH)D and iCa as given

Over the 5 years, SHPT differed between subgroups of S-25(OH)D (Fig. 2). Higher S-25(OH)D levels were associated with lower occurrence of SHPT: OR 0.44 (0.36–0.54) with S-25(OH)D ≥ 50 nmol/l, OR 0.38 (0.30–0.49) with S-25(OH)D ≥ 75 nmol/l, OR 0.19 (0.12–0.31) with S-25(OH)D ≥ 100 nmol/l, all compared with S-25(OH)D < 50 nmol/l. Compared with S-25(OH)D 50–99 nmol/l, OR was 0.40 (0.26–0.62) with S-25(OH)D ≥ 100 nmol/l.

**Fig. 3:**
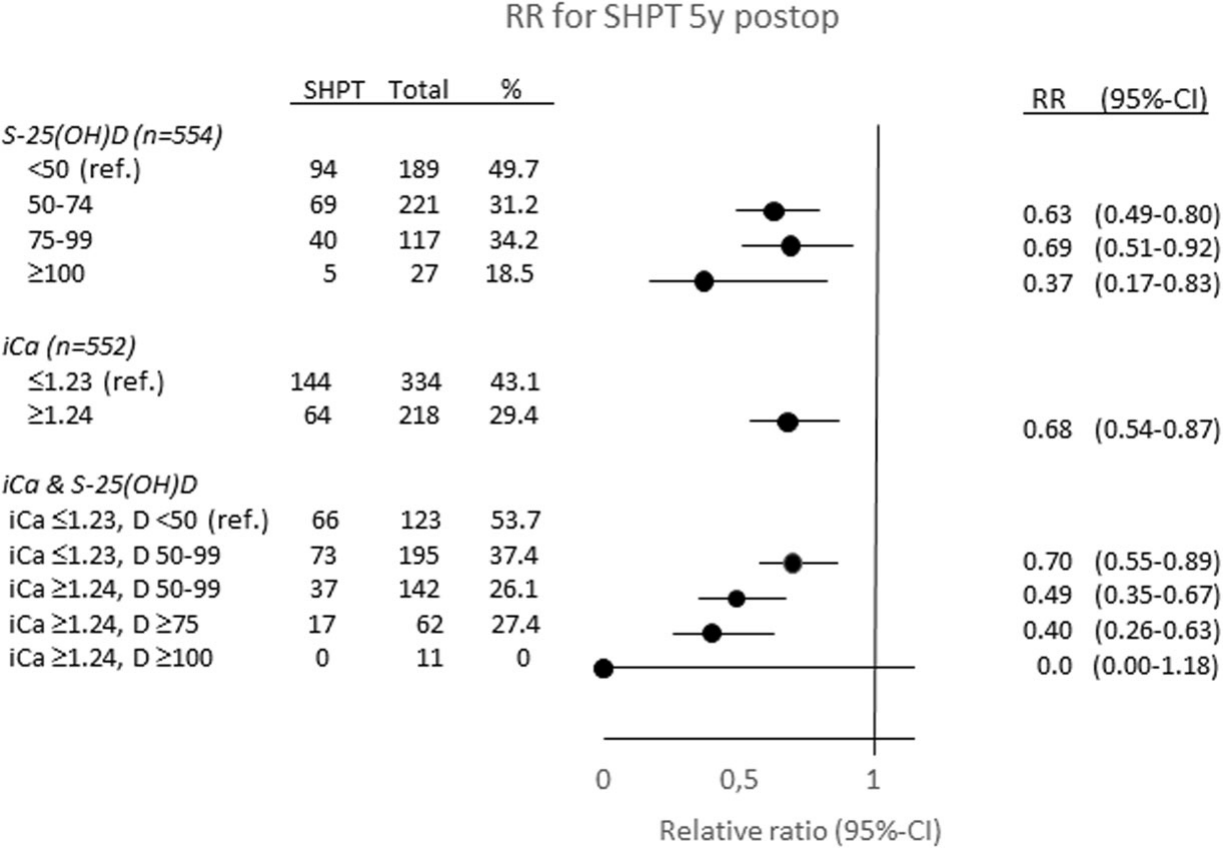
Relative ratio (RR) for secondary hyperparathyroidism (SHPT) 5 years after Roux-en-Y gastric bypass (*N* = 554) by categories of serum 25-hydroxyvitamin D (S-25(OH)D or D, nmol/l) and ionized calcium (iCa, mmol/l) compared with reference (ref.)

At 5 years, 208 of 554 patients (38%) had SHPT. The prevalence was 50% with S-25(OH)D < 50 nmol/l (Fig. 3/Table 2); 61% with S-25(OH)D < 25 nmol/l. SHPT was less frequent with S-25(OH)D ≥ 50 nmol/l (31%, RR 0.63 (0.51–0.77)), S-25(OH)D ≥ 75 nmol/l (31%, RR 0.63 (0.47–0.83)), and lowest with S-25(OH)D ≥ 100 nmol/l (19%, RR 0.37 (0.17–0.83)), all compared with S-25(OH)D < 50 nmol/l. Compared with S-25(OH)D 50–99 nmol/l, PTH was lower with S-25(OH)D ≥ 100 nmol/l (5.0 ± 2.1 compared with 6.4 ± 3.3 pmol/l, *P* = 0.032), but SHPT not significantly (RR 0.57 (0.25–1.28)) at 5 years. These relationships were not significant with iCa above lower range (iCa ≥1.24 mmol/l).

**Table 2:** Observed numbers with secondary hyperparathyroidism by vitamin D status at follow-up from 6 months to 5 years after Roux-en-Y gastric bypass (*N* = 554)

	6 months		1 year		2 years		3–4 years		5 years	
	SHPT	All	SHPT	All	SHPT	All	SHPT	All	SHPT	All
S-25(OH)D										
< 50	25	77	38	100	57	121	39	86	94	188
50–74	20	125	49	178	57	171	50	147	69	222
75–99	17	126	35	134	28	117	20	72	40	117
≥ 100	6	51	2	39	3	30	2	14	5	27
All	68	379	124	451	145	439	111	319	208	554
Missing		175		103		115		235		0

*S-25(OH)D*, serum 25-hydroxyvitamin D; *SHPT*, secondary hyperparathyroidism; *All*, attenders

### Ionized Calcium and BMI

**Fig. 4:**
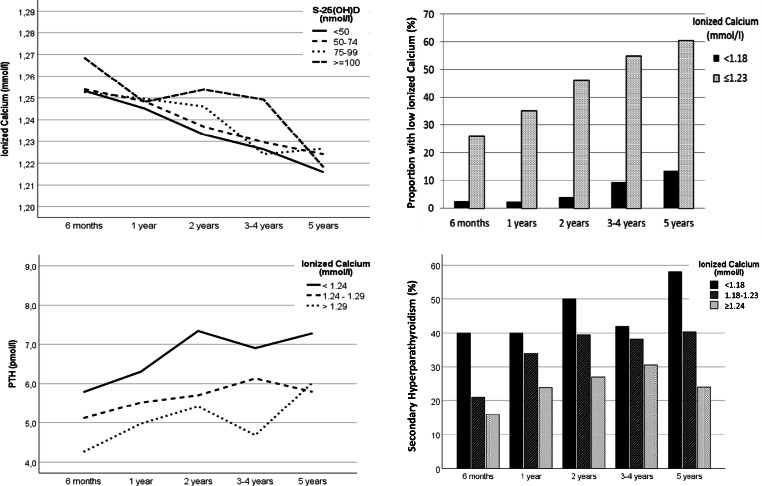
Observed development of ionized calcium by subgroups of 25-hydroxyvitamin D (S-25(OH)D), and proportions of low calcium and calcium within the lower range from 6 months to 5 years after Roux-en-Y gastric bypass (*N* = 552) (upper), and development of parathyroid hormone (PTH) and proportion with secondary hyperparathyroidism by subgroups of ionized calcium (lower)

Serum iCa decreased in all subgroups of vitamin D from 6 months to 5 years postoperatively, and percentage with iCa in the lower range (iCa ≤1.23) increased from 26% to 61% (*P* < 0.001) (Fig. 4). PTH increased in all 3 subgroups of iCa over 5 years, and was higher in the lower range. iCa was inversely related with PTH development (P < 0.001). Correspondingly, SHPT was consistently lower with iCa above lower range during follow-up.

An interaction existed between S-25(OH)D and iCa. At 5 years, SHPT was most prevalent with combined vitamin D deficiency and iCa in the lower range, and there was no SHPT with S-25(OH)D ≥ 100 nmol/l and iCa above the lower range (Fig. 3).

### Multivariate Analyses

In multivariate analyses of PTH from 6 months to 5 years (Table 3), the relationship with S-25(OH)D and iCa remained robust, also with BMI. Interactions for S-25(OH)D and iCa with time over the 5 years were not significant in multivariate analyses. SHPT occurred more often in men, with lower age and with higher BMI over the period (Supplementary Table). The trend of lower OR with higher S-25(OH)D remained across strata of gender, age and BMI.

**Table 3:** Regression analysis^a^ of parathyroid hormone and modulating factors from 6 months to 5 years after Roux-en-Y gastric bypass (*N* = 554)

	*B*	SE	*P*
Univariate			
S-25(OH)D	− 0.032	0.0025	< 0.001
iCa	− 23.9	1.46	< 0.001
BMI	0.12	0.014	< 0.001
Phosphate	− 3.57	0.38	< 0.001
Magnesium	− 0.12	0.031	0.69
Creatinine	0.023	0.0075	0.003
Time	0.32	0.028	< 0.001
Preop Vit D	− 0.034	0.0091	< 0.001
Gender^b^	0.35	0.23	0.13
Age^c^	− 0.0069	0.012	0.56
Multivariate^d^			
I	42.2	4.86	< 0.001
S-25(OH)D	− 0.248	0.064	< 0.001
iCa	− 30.65	3.81	< 0.001
BMI	0.065	0.014	< 0.001
S-25(OH)D × iCa	0.182	0.051	< 0.001
Creatinine	0.020	0.0076	0.009
Time	0.073	0.030	0.015

^a^Linear mixed model; ^b^men 1, women 0; ^c^Age at 5 years; ^d^Adjusted for age and gender; ×, interaction

*RYGB*, Roux-en-Y gastric bypass; *I*, intercept; *B*, regression coefficient; *SE*, standard error; *S-25(OH)D*, serum 25-hydroxyvitamin D (nmol/l); *iCa*, ionized calcium (mmol/l); *BMI*, body mass index (kg/m^2^);

Creatinine (μmol/l); Time, time postoperatively (years); Preop Vit D, S-25(OH)D preoperatively

### Supplementation, PTH/SHPT and Bone Turnover

360 of 475 (77%) used supplements of calcium and vitamin D at 6 months, and 278 (50%) at 5 years. S-25(OH)D was higher and PTH lower in users over the period (*P* < 0.001), but not significantly at 5 years.

PTH related positively with B-ALP and ALP over the 5 years (P < 0.001), with interaction for PTH and time. B-ALP declined by 1.3 U/l per year, but remained increased with SHPT (P < 0.001 for group difference). Total ALP declined slower with SHPT (*P* = 0.004).

## Discussion

PTH and the prevalence of SHPT decreased the first 6 months after RYGB, and thereafter increased up to 5 years. S-25(OH)D was related with PTH development. Patients with vitamin D deficiency had the largest increase in PTH from 6 months, while patients with high S-25(OH)D ≥ 100 nmol/l had lowest PTH and prevalence of SHPT.

### Vitamin D and SHPT

This study is among few longitudinal long-term reports of SHPT by vitamin D and calcium status after RYGB. Vitamin D deficiency is usually defined by S-25(OH)D < 30–50 nmol/l, while target levels of S-25(OH)D ≥ 50–75 nmol/l are adopted in most recommendations after obesity surgery [1, 2, 4–8]. However, evidence supporting these target levels is limited [22, 24].

The observed increase in PTH from 6 months is in accordance with other long-term evaluations [9–13]. S-25(OH)D was strongly related with PTH over time, however, with limited differences in SHPT by traditional target thresholds, defined by S-25(OH)D ≥ 50 nmol/l and ≥ 75 nmol/l. The findings with S-25(OH)D ≥ 100 nmol/l corresponded with our cross-sectional study 2 years postoperatively [20].

### Calcium and SHPT

Calcium absorption seems reduced after RYGB [31, 32]. However, few have reported a relationship between calcium and PTH [18, 20, 23]. Extracellular calcium is a determinant of PTH secretion, and even calcium within the lower normal range may increase PTH [20, 33, 34]. With the feedback mechanisms involved, PTH may increase above reference range, while serum calcium still remains within normal reference range.

Our observations suggest a role of iCa on PTH. The proportion of patients with iCa in the lower range increased over time, and iCa was related with PTH development. This relationship was independent of S-25(OH)D. An interaction existed between S-25(OH)D and iCa on PTH. Still, iCa declined in all subgroups of S-25(OH)D but more slowly with higher levels.

### Supplementation, SHPT and Bone Effects

Several studies have failed to document benefits of vitamin D and calcium supplementation, and the regimens have been questioned [22, 24]. This study supports a modest effect with supplements of calcium ≥ 500 mg and vitamin D ≥ 600 IU on PTH. These doses are however lower than recommended by many [2, 4, 6].

We also found higher B-ALP in patients with SHPT up to 5 years postoperatively, suggesting higher bone turnover. SHPT might therefore help explain increased bone turnover, which is observed up to 5 years after RYGB [18, 19, 22]. SHPT may also lead to reduced BMD, which is observed after weight stabilizes 1–2 years postoperatively [15, 19]. We recently found SHPT related with lower BMD 10 years after RYGB [21].

### Implications

In clinical practice, SHPT may be considered as a marker of vitamin D and calcium insufficiency, and it is of concern after RYGB. Further research should address whether increasing S-25(OH)D levels can suppress SHPT and improve bone health. Higher doses seem necessary to achieve sufficient vitamin D levels and suppress SHPT after RYGB [2, 4, 24, 35].

Attention to calcium status seems relevant to identify risk for SHPT. SHPT was more frequent with iCa in the lower range. Higher S-25(OH)D can increase calcium levels and lower PTH, and some individuals may need higher vitamin D levels than others [20, 26, 36]. The interaction between S-25(OH)D and iCa may be relevant in clinical practice. SHPT was not prevalent with S-25(OH)D ≥ 100 nmol/l and iCa in the upper two tertiles of reference range at 5 years, which we previously reported 2 years postoperatively [20].

Optimal S-25(OH)D levels are not established after RYGB and obesity surgery in general. Achieving S-25(OH)D ≥ 100 nmol/l may be needed to suppress SHPT more effectively in some individuals, with an aim to improve long-term bone health.

### Strengths and Limitations

The main strength of this study was a large sample size with high 5-year follow-up rate and repeated measurements, providing statistical strength. Bias of primary hyperparathyroidism was minimized. The single centre study design with standard surgical and follow-up procedures strengthen internal validity, but the findings need testing in other populations. As relationships up to 5 years were the primary focus, only patients with 5-year data on S-25(OH)D and PTH were included. Prevalence of SHPT in nonattenders may be higher than observed, assuming less compliance [37]. Data on preoperative S-25(OH)D and PTH, and S-25(OH)D ≥ 100 nmol/l and B-ALP, during follow-up were limited. Supplemental use was self-reported. iCa determinations at the Hormone Laboratory were adjusted during the period; however, the drop in iCa was also found in some parallel analyses performed at the Central Laboratory, with unchanged methodology.

## Conclusions

S-25(OH)D levels related inversely with PTH development and occurrence of SHPT up to 5 years after RYGB. The prevalence of SHPT was lower with S-25(OH)D thresholds ≥ 50 nmol/l and ≥ 75 nmol/l. Some patients may need S-25(OH)D ≥ 100 nmol/l to suppress SHPT more effectively.

## Electronic supplementary material

ESM 1 (DOCX 22 kb)
