# Supplementary material for: Relationships Between Vitamin D Status and PTH over 5 Years After Roux-en-Y Gastric Bypass: a Longitudinal Cohort Study
Source: Obes Surg. 2020 Apr 18;30(9):3426–34. doi: 10.1007/s11695-020-04582-5 (PMC7378105; doi:10.1007/s11695-020-04582-5)
Supplement: Supplementary file 1 — (DOCX 22 kb) [file 11695_2020_4582_MOESM1_ESM.docx]

| **Supplementary Table.** Observed numbers and occurrence of Secondary Hyperparathyroidism in 554 Roux-en-Y Gastric Bypass patients from 6 months to 5 years postoperatively by groups of gender, age and BMI. | | | | | | | | | | | | | |  |
| --- | --- | --- | --- | --- | --- | --- | --- | --- | --- | --- | --- | --- | --- | --- |
|  |  |  |  |  |  |  |  |  |  |  |  |  |  | |
|  |  |  | 6m |  | 1y |  | 2y |  | 3-4y |  | 5y |  | Total | |
| *Gender* |  |  |  |  |  |  |  |  |  |  |  |  |  | |
|  |  |  |  |  |  |  |  |  |  |  |  |  |  | |
| Men | SHPT (n) |  | 24 |  | 45 |  | 48 |  | 34 |  | 75 |  | 226 | |
|  | No SHPT (n) |  | 90 |  | 94 |  | 80 |  | 59 |  | 95 |  | 418 | |
|  | % SHPT |  | 21.1 |  | 32.4 |  | 37.5 |  | 36.6 |  | 44.1 |  | 35.1 | |
|  |  |  |  |  |  |  |  |  |  |  |  |  |  | |
| Women | SHPT (n) |  | 44 |  | 82 |  | 97 |  | 77 |  | 133 |  | 433 | |
|  | No SHPT (n) |  | 226 |  | 237 |  | 216 |  | 149 |  | 251 |  | 1079 | |
|  | % SHPT |  | 16.3 |  | 25.7 |  | 31.0 |  | 34.1 |  | 34.6 |  | 28.6 | |
|  |  |  |  |  |  |  |  |  |  |  |  |  |  | |
| *Age (y)* |  |  |  |  |  |  |  |  |  |  |  |  |  | |
|  |  |  |  |  |  |  |  |  |  |  |  |  |  | |
| <40 | SHPT (n) |  | 14 |  | 18 |  | 30 |  | 24 |  | 49 |  | 135 | |
|  | No SHPT (n) |  | 58 |  | 72 |  | 54 |  | 34 |  | 66 |  | 284 | |
|  | % SHPT |  | 19.4 |  | 20.0 |  | 35.7 |  | 41.4 |  | 42.6 |  | 32.2 | |
|  |  |  |  |  |  |  |  |  |  |  |  |  |  | |
| 40-50 | SHPT (n) |  | 31 |  | 75 |  | 69 |  | 51 |  | 89 |  | 315 | |
|  | No SHPT (n) |  | 138 |  | 136 |  | 139 |  | 92 |  | 164 |  | 669 | |
|  | % SHPT |  | 18.3 |  | 35.5 |  | 33.2 |  | 35.7 |  | 35.2 |  | 32.0 | |
|  |  |  |  |  |  |  |  |  |  |  |  |  |  | |
| >50 | SHPT (n) |  | 23 |  | 34 |  | 46 |  | 36 |  | 70 |  | 209 | |
|  | No SHPT (n) |  | 120 |  | 123 |  | 103 |  | 82 |  | 116 |  | 544 | |
|  | % SHPT |  | 16.1 |  | 21.7 |  | 30.9 |  | 30.5 |  | 37.6 |  | 27.8 | |
|  |  |  |  |  |  |  |  |  |  |  |  |  |  | |
| *BMI (kg/m2)* |  |  |  |  |  |  |  |  |  |  |  |  |  | |
|  |  |  |  |  |  |  |  |  |  |  |  |  |  | |
| <30.0 | SHPT (n) |  | 13 |  | 31 |  | 49 |  | 29 |  | 38 |  | 160 | |
|  | No SHPT (n) |  | 65 |  | 151 |  | 116 |  | 65 |  | 94 |  | 491 | |
|  | % SHPT |  | 16.7 |  | 17.0 |  | 29.7 |  | 30.9 |  | 28.8 |  | 24.6 | |
|  |  |  |  |  |  |  |  |  |  |  |  |  |  | |
| 30.0-34.9 | SHPT (n) |  | 26 |  | 50 |  | 40 |  | 32 |  | 54 |  | 202 | |
|  | No SHPT (n) |  | 126 |  | 110 |  | 107 |  | 72 |  | 135 |  | 550 | |
|  | % SHPT |  | 17.1 |  | 31.3 |  | 27.2 |  | 30.8 |  | 28.6 |  | 26.9 | |
|  |  |  |  |  |  |  |  |  |  |  |  |  |  | |
| 35.0-39.9 | SHPT (n) |  | 15 |  | 25 |  | 41 |  | 30 |  | 68 |  | 179 | |
|  | No SHPT (n) |  | 64 |  | 39 |  | 44 |  | 41 |  | 72 |  | 260 | |
|  | % SHPT |  | 19.0 |  | 39.1 |  | 48.3 |  | 42.3 |  | 48.6 |  | 40.8 | |
|  |  |  |  |  |  |  |  |  |  |  |  |  |  | |
| ≥40 | SHPT (n) |  | 10 |  | 15 |  | 12 |  | 13 |  | 48 |  | 98 | |
|  | No SHPT (n) |  | 43 |  | 18 |  | 15 |  | 20 |  | 44 |  | 140 | |
|  | % SHPT |  | 18.9 |  | 45.5 |  | 44.4 |  | 39.4 |  | 52.2 |  | 41.2 | |
| SHPT, Secondary hyperparathyroidism; Age (y), age 5 years postoperatively; BMI, body mass index. | | | | | | | | | | | | | |  |
